# Seroprevalence of Hand, Foot and Mouth Disease Among Children and Adolescents in Türkiye

**DOI:** 10.3390/vaccines14060470

**Published:** 2026-05-25

**Authors:** Adem Karbuz, Tuğce Tural-Kara, Ümit Çelik, Belgin Gülhan, Ayşegul Elvan-Tüz, Yasemin Coşgun, Çigdem Kirmaci, Ayşe Kübra Açık, Merve Kılıç-Çil, Saliha Kanık-Yüksek, Dilek Yılmaz-Çiftdoğan, Merve Zerey-Albayrak, Vildan Şahin, Tuğba Erat, Şilem Özdem-Alataş, Ekrem Sağtaş, Erdem Öksüzoğlu, Muhammed Emin Demirkol, Ateş Kara

**Affiliations:** 1 Pediatric Infectious Diseases Clinic, Prof. Dr. Cemil Taşcıoglu City Hospital, 34384 Istanbul, Türkiye; 2 Department of Pediatric Infectious Diseases, Cerahpaşa Medical Faculty, Istanbul University Cerrahpaşa, 34320 Istanbul, Türkiye; 3 Department of Pediatric Infectious Diseases, Faculty of Medicine, Akdeniz University, 07070 Antalya, Türkiye; 4 Pediatric Infectious Diseases Clinic, Adana City Hospital, 01230 Adana, Türkiye; 5 Pediatric Infectious Diseases Clinic, Ankara Bilkent City Hospital, 06800 Ankara, Türkiye; 6 Pediatric Infectious Diseases Clinic, İzmir City Hospital, 35540 Izmir, Türkiye; 7 Virology Reference Laboratories, General Directorate of Public Health, Republic of Türkiye Ministry of Health, 06100 Ankara, Türkiye; 8 Department of Child Health and Diseases, Faculty of Medicine, İzmir Katip Çelebi University, 35620 Izmir, Türkiye; 9 Pediatric Infectious Diseases Clinic, University of Health Sciences, Izmir Tepecik Training and Research Hospital, 35020 Izmir, Türkiye; 10 Directorate of Public Health, Republic of Türkiye Ministry of Health, 06100 Ankara, Türkiye; 11 Department of Pediatrics, Pediatric Infectious Diseases, Faculty of Medicine, Hacettepe University, 06100 Ankara, Türkiye; 12 Türkiye Vaccine Institute, 06270 Ankara, Türkiye

**Keywords:** hand, foot, and mouth disease, seroprevalence, EV-A71, CV-A16, CV-A10, CV-A6, risk factors, children, adolescents, Türkiye

## Abstract

**Background/Objectives**: Hand, foot and mouth disease (HFMD) has recently emerged as a serious health threat, as certain serotypes can cause severe illness. Serotype distribution vary by region, and seroprevalence studies helps in developing preventive strategies. This study aimed to determine the seroprevalence of enterovirus type 71 (EV-A71), Coxsackievirus A16 (CV-A16), Coxsackievirus A10 (CV-A10), and Coxsackievirus A6 (CV-A6), the main causative agents of HFMD and to investigate risk factors for seropositivity. **Methods**: This multicenter, cross-sectional study was conducted across five major cities in Türkiye. Children (6 months–17 years) who presented to outpatient clinics for any reason were included between May 2024 and January 2025. Neutralizing antibodies were measured using a microneutralization assay. Statistical analyses included descriptive methods, appropriate group comparisons (Chi-square/Fisher’s Exact), and backward logistic regression to identify factors associated with HFMD seropositivity. **Results**: The study included 998 participants (mean age: 8.6 ± 5.2 years; 51.3% male). CV-A6 antibodies were detected in 68.5%, EV-A71 in 66.5%, CV-A10 in 60.2%, and CV-A16 in 46.0% of samples. No viral antibodies were detected in 5.3% of serum samples (All-Negative group); antibodies against at least one HFMD agent were detected in 94.7% (Any-Positive group). HFMD seropositivity increased significantly with age. Handwashing habits did not differ between the groups. The any-positive group more often had a household member aged 12–18 years, a mother with lower education, and higher kindergarten attendance. In logistic regression analysis, age, average monthly household income, and mother’s education level were the factors influencing seropositivity. **Conclusions**: The seroprevalence of HFMD-causing viruses in Türkiye is high from six months of age onward. Beyond promoting personal protective measures, the implementation of a vaccination program should also be considered.

## 1. Introduction

Hand-foot-and-mouth disease (HFMD) is a common contagious illness primarily affecting children under the age of five, though older children and adults can also be infected. HFMD is characterized by a papulovesicular or maculopapular rash, blisters on the hands, soles, and buttocks, and painful ulcerative lesions in the mouth [1,2]. It is generally self-limited, but a small proportion of children may experience severe complications such as meningitis, encephalitis, acute flaccid paralysis, and cardiopulmonary failure. In the past few decades, epidemiological and clinical studies have revealed that the disease may be associated with potentially fatal complications, thereby altering the initial understanding and drawing greater attention to HFMD [1,2]. Recently, HFMD has emerged as a serious health hazard, especially in Asia-Pacific countries. HFMD outbreaks, seen in many countries worldwide, have reported serious complications and deaths [3].

Currently, there is no specific treatment for HFMD. Symptomatic and supportive treatment is applied, patients are isolated to prevent cross-infection, and good oral and skin care is provided [3]. Vaccination is considered the most effective and cost-effective approach to controlling the incidence of HFMD. Currently, monovalent and polyvalent vaccines against the HFMD pathogen are available, and vaccine studies are ongoing [3,4].

HFMD is caused by enteroviruses (EVs), which belong to the genus *Enterovirus* within the family *Picornaviridae*. The most frequently implicated pathogens are members of the *Enterovirus A* (EV-A) species. Among the various EV-A species associated with HFMD, the most known are enterovirus type 71 (EV-A71) and Coxsackievirus A16 (CV-A16). HFMD due to other viruses such as Coxsackievirus A10 (CV-A10) and Coxsackievirus A6 (CV-A6) has also been reported [2]. HFMD is typically diagnosed clinically without microbiological tests. However, identifying the specific viral serotype is crucial, as certain serotypes can cause severe illness and serotype distribution may vary across regions. Increased global travel may contribute to the spread of different serotypes. Effective and continuous surveillance, or at least the identification of circulating serotypes through seroepidemiological studies, is essential in combating the disease. Accurate identification of causative serotypes supports public health efforts, informs preventive strategies, and aids vaccine development [4]. In this context, this study aimed to determine the seroprevalence of EV-A71, CV-A16, CV-A10, and CV-A6, which are the main causative agents of HFMD, and to investigate risk factors for seropositivity among children and adolescents in Türkiye. Thus, we believe that determining the frequency of exposure to these agents in our country will provide valuable data to policymakers regarding vaccination strategies.

## 2. Materials and Methods

### 2.1. Design

This multicenter, cross-sectional study was conducted in five hospitals across five major cities in Türkiye (Ankara, Antalya, Adana, İzmir, and İstanbul) between May 2024 and January 2025. The characteristics of the study areas are summarized in Appendix A Table A1.

**Figure 1:**
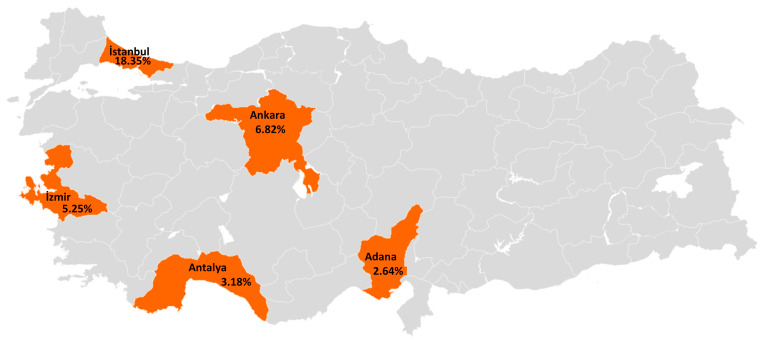
Study center locations and their share of the national population. Note: Percentages indicate each city’s share of the national population.

Hospital-based recruitment was selected to facilitate data collection from participants who met the eligibility criteria. Cities were selected to optimize geographical representation, and study hospitals were chosen based on their strong willingness to participate, as well as their capability and resources to conduct the study. The locations of study centers in Türkiye are shown in Figure 1. The cities where the study was conducted account for about one-third of the country’s population (36.25%).

**Figure A1:**
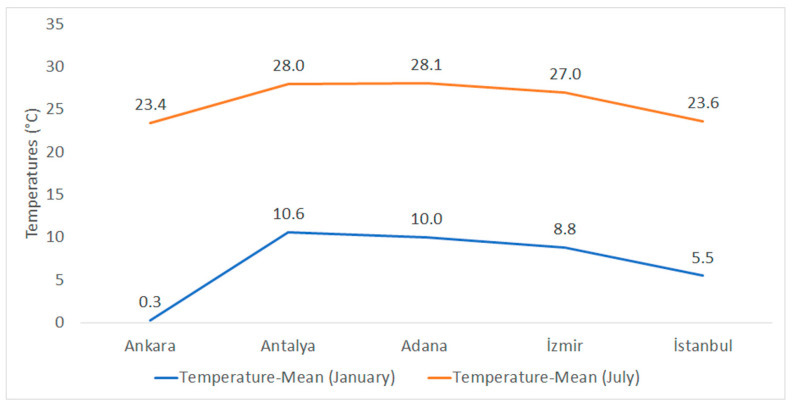
The mean summer (July) and winter (January) temperatures (°C) of the cities.

The mean summer (July) and winter (January) temperatures of the relevant cities are presented in Appendix A in Figure A1. The highest temperatures in summer are in Adana, and the lowest temperatures in winter are in Ankara.

### 2.2. Participants

Children aged 6 months to 17 years who presented to outpatient clinics for any reason and required blood collection were included in the study. Individuals who had difficulty obtaining blood samples due to previous conditions such as coagulation abnormalities or hematomas, or petechiae after intramuscular injections or venipuncture, patients with autoimmune disease or immunodeficiency/immunosuppression (including but not limited to congenital immunodeficiency, systemic lupus erythematosus, ankylosing spondylitis, autoimmune thyroid disease, anaplasmosis, absent functional spleen, Human Immunodeficiency Virus [HIV] infection), patients with serious neurological disorders (e.g., epilepsy, convulsions, or seizures), psychiatric diseases or a family history of psychiatric disorders, those who had participated in any other clinical trial within the last six months, those who had received immunosuppressive or other immunomodulatory therapy within the last year, or those who had used immunoglobulin or other blood products within the last year were excluded. Participants were required to be available throughout the study period.

In Türkiye, healthcare services are provided free of charge for individuals under 18 years of age; therefore, no direct payment was required from the families of the participating children. In addition, no promotional or incentive-based recruitment strategies were employed, and participation was entirely voluntary.

The study was approved by the Clinical Research Ethics Committee of Ankara Bilkent City Hospital (Approval Number: E2-24-6875; Date: 20 March 2024). Written informed consent was obtained from the participant or parent/guardian/legal representative.

### 2.3. Procedure

Information on participants who met the inclusion and exclusion criteria was recorded in case report forms (Supplementary File S1: Case Report Form of the Study in Turkish). Blood samples collected in clotted blood tubes were centrifuged 2000–4000× *g* for 15 min at room temperature. The resulting sera were aliquoted into two clearly labeled cryovials, each containing approximately 500 microliters of serum, designated as the main sample and backup sample. Serum samples were stored at −20 °C or below until testing. All samples were transferred to the central laboratory (Turkish Ministry of Health, General Directorate of Public Health National Reference Laboratory) for analysis. Neutralizing antibodies against EV-A71, CV-A16, CV-A10, and CV-A6 were measured using a microneutralization assay. Seropositivity was defined as a neutralizing antibody titer of ≥1:8. Laboratory studies were conducted in accordance with the standard operating procedures of the World Health Organization [5].

### 2.4. Statistical Analysis

The sample size was calculated assuming that the overall seroprevalence of neutralizing antibodies against EV-A71 is 20%. Based on a 95% confidence interval (CI) with a width of 5%, a sample size of 1022 participants was calculated using PASS 15.0 (NCSs, LLC. Kaysville, UT, USA). Considering other factors (such as time or logistical limits), around 1000 participants were planned to be enrolled. Thus, the study aimed to recruit 200 participants from each center, with 50 individuals allocated to each of the following age groups: 6 months–2 years, 3–5 years, 6–11 years, and 12–17 years.

Statistical analysis was performed using PASW 18.0 for Windows (SPSS, Inc., Chicago, IL, USA). Descriptive statistics were presented as numbers and percentages for categorical variables, and as means and standard deviations for numerical variables. The normality of data was tested using visual (histogram and probability graphs) and analytical methods (Kolmogorov–Smirnov/Shapiro–Wilk tests). For comparisons of categorical variables between two or multiple groups, Fisher’s Exact test was used when the assumptions of the Chi-Square test were not met. To determine the factors affecting seropositivity for HFMD, a logistic regression analysis was performed using the backward method on a model created with clinically and/or statistically significant variables (*p* < 0.200). Statistical significance level was accepted as *p* < 0.05.

## 3. Results

The study included 998 participants from 5 centers. The mean age of the participants was 8.6 ± 5.2, and 51.3% were male. General characteristics of the participants are shown in Table 1.

**Figure 2:**
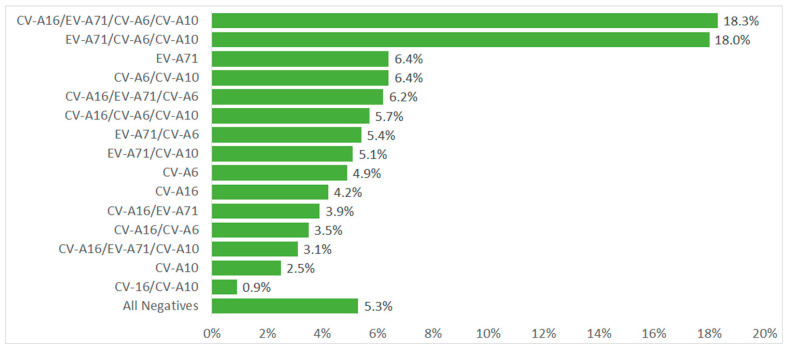
Viral antibody test results.

Viral antibody test results are shown in Figure 2. No viral antibodies were detected in 53 (5.3%) of the serum samples. Single viral antibodies were detected in 180 (18.0%) samples, dual in 252 (25.3%) samples, triple in 330 (33.1%) samples, and quadruple in 183 (18.3%) samples.

Viral antibody test results by age group are presented in Table 2. As age increased, a significant increase was observed in the likelihood of exposure to viruses associated with HFMD. The frequency of quadruple viral antibodies was found to be 4.8% in the 6–8-month age group and 25.3% in the 12–17-year age group.

**Figure 3:**
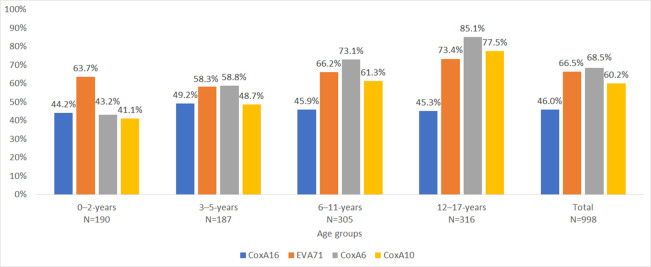
Seroprevalence of EV-A71, CV-A16, CV-A10, and CV-A6 in all participants and by age group.

When viruses were considered separately, it was observed that CV-A6 antibodies were detected in 684 (68.5%) of the samples, EV-A71 in 664 (66.5%), CV-A10 in 601 (60.2%), and CV-A16 antibodies in 459 (46.0%). The seroprevalence of EV-A71, CV-A16, CV-A10, and CV-A6 among all participants and by age group is presented in Figure 3.

The distribution of viral antibody-positive samples by age group is shown in Table 3. CV-A16 antibody positivity was found at similar rates across age groups. EV-A71, CV-A6, and CV-A10 antibody positivity was found to be highest in the 12–17 age group.

No significant difference was found between centers in terms of CV-A6 and CV-A10 antibody positivity. The highest CV-A16 antibody positivity rate was found in Adana (66.5%) and the lowest in Istanbul (24.2%). EV-A71 antibody positivity was found to be highest in Ankara (74.2%) and the lowest in Istanbul (53.4%) (Table 3).

Serotype distribution by age groups and study sites is shown in detail in Appendix A Table A2.

The participants showing no antibodies against any HFMD pathogen (All-Negative group, n = 53) were compared with those showing antibodies against any HFMD pathogen (Any-Positive group, n = 945) in terms of certain characteristics. A significant increase in HFMD seropositivity was found with increasing age. Handwashing habits did not differ between the two groups. The any-positive group had a higher frequency of having a household member aged 12–18 years and of having a mother with a lower education level. Attending kindergarten was significantly higher in the any-positive group (Table 4).

In the logistic regression analysis, a model was created using the variables of age, sex, nursery attendance, kindergarten attendance, household’s average monthly income, mother’s and father’s education level. Factors influencing seropositivity were identified as follows: a 1-unit increase in age (1.177 times), a household’s average monthly income between 50,000 and <75,000 TL (4.056 times), a household’s average monthly income ≥75,000 TL (5.640 times), and a mother being a high school graduate (3.419 times) (Table 5).

## 4. Discussion

The first case of HFMD was identified in 1948, and in the 1970s, outbreaks of EV-associated HFMD were reported in many regions worldwide. In the 1990s, outbreaks of EV-associated HFMD, accompanied by complications, permanent sequelae, and deaths, were reported from Asian countries. Outbreaks continue to be reported from many regions from the 2000s to the present [6]. In the past, HFMD received little attention, as it was considered a self-limiting disease. However, with increasing reports of outbreaks and cases involving serious complications, it has emerged as a significant public health concern [6]. Identifying the circulating HFMD agents is important, as certain serotypes, especially EV-A71, are associated with severe outcomes [7]. When recent outbreaks are examined, it has been noted that EV-A71 and CV-A16 tend to be gradually replaced by CV-A6 and CV-A10 as the main pathogens of HFMD, and the incidence of CV-B3 and CV-B5 infections is increasing [6]. Continuous monitoring of this changing trend in the causative serotypes is necessary to take effective preventive measures for HFMD infection [8]. Seroprevalence studies reveal past infections and help identify the prevalent serotypes in a given area. These data also inform the selection of candidate serotypes for vaccine development. Among recent studies, a seroprevalence study conducted in 220 children (7 months–15 years old) in Korea found EV-A71 and CV-A6 to be suitable candidate serotypes for vaccine development, while further studies were needed for CV-A10 and CVA-16. The highest seropositivity in all age groups was detected for CV-A6 (73.6%), followed by EV-A71, (64.1%), CV-A10 (47.7%), and CV-A16 (34.5%) [9]. Similarly, the highest seropositivity was observed for CV-A6 (65.80%), followed by EV-A71, (59.61%), CV-A10 (58.96%), and CV-A16 (40.07%) in a seroprevalence study conducted in China among healthy individuals (n = 307) across various age groups [10]. Analysis of HFMD cases reported in China between 2009 and 2023 confirmed that CV-A6 gradually emerged as the dominant serotype, while the prevalence of EV-A71 and CV-A16 declined over time [11]. In a seroprevalence study conducted in the United Kingdom, the overall seropositivity rate was determined as 80% for CV-6 and 74% for EV-A71 in archive serum samples from all age groups from 2006 (n = 514), 2011 (n = 498), and 2017 (n = 561) [12]. On the other hand, in a seroprevalence study involving 600 children aged 6–71 months in Indonesia, EV-A71 immunoglobulin G (IgG) positivity was found to be quite high (99.3%) [13].

In Türkiye, the published manuscripts on HFMD have generally focused on the clinical aspects of the disease or on case reports [14,15,16,17,18,19]. A study conducted in Istanbul analyzed 27 patient samples collected between 2015 and 2017. Laboratory testing using RT-PCR revealed the presence of enterovirus in 12 samples. Among these, CV-A16 was detected in 3 cases and CV-A6 in 9, supporting the observation that CV-A6-related cases are increasing in frequency [20]. A seroprevalence study conducted among preschool children (n = 380) in Sakarya showed the EV-A71 IgG antibody positivity was 57.9% and CV-A16 IgG antibody positivity was 57.4% [21]. Our study, conducted in different regions of Türkiye, is the first of its kind to conduct a comprehensive seroprevalence study. Neutralizing antibodies against at least one HFMD agent were detected in 94.7% of serum samples collected from 998 children aged 6 months to 17 years. Antibodies to CV-A6 (68.5%) and EV-A71 (66.5%) were the most prevalent, followed by CV-A10 (60.2%) and CV-A16 (46.0%). Our findings are consistent with global seroprevalence patterns observed in recent studies mentioned above.

Seropositivity rates were evaluated separately in the 6 month–2 year, 3–5-year, 6–11-year, and 12–17-year age groups. CV-A16 seropositivity remained relatively consistent across all age groups, whereas the highest antibody positivity rates for EV-A71, CV-A6, and CV-A10 were observed in the 12–17-age group. This age-related increase in antibody prevalence likely reflects cumulative exposure, as HFMD primarily occurs in early childhood. On the other hand, most participants had more than one antibody detected, and when all serotypes were considered together, the rate of at least one antibody positivity was 90.5% (n/N = 19/21) even in children aged 6–8 months. Comparing individuals seronegative for HFMD with those who had at least one antibody positivity for any HFMD, the seroprevalence rate was shown to increase with age. Of the any-positive individuals (n = 945), 2% were in the 6–8-month age group, while 33.1% were in the 12–17-year age group (*p* < 0.001).

There are environmental factors that affect disease epidemiology. Climatic characteristics such as ambient temperatures and humidity have found to be associated with the incidence of HFMD [22,23,24,25,26]. When seroprevalence rates were examined by region in our study, the highest CV-A16 antibody positivity rate was found in Adana (66.5%) and the lowest in Istanbul (24.2%). The highest EV-A71 antibody positivity rate was found in Ankara (74.2%) and the lowest in Istanbul (53.4%). No difference was found between regions in terms of CV-A6 and CV-A10 seroprevalence. Our findings have confirmed that certain HFMD-associated agents exhibit variable frequencies of occurrence, which appear to be influenced by regional and climatic factors. Socioeconomic factors are also closely related to the epidemic and severity of HFMD [27,28]. In our study, the HFMD seropositive group had a higher frequency of having a household member aged 12–18 years and of having a mother with a lower education level.

Due to the absence of a specific treatment for HFMD and the diversity of its causative agents -with reinfection remaining a possibility- preventive measures are essential for effective disease control [29]. Enteroviruses, the principal pathogens responsible for HFMD, are thought to be transmitted primarily via the fecal-oral route, respiratory droplets and aerosols, or through close personal contact [6]. Personal hygiene, hand washing, household crowding, and nutritional habits are associated with the risk of HFMD [6,30,31,32]. For these reasons, personal hygiene and improving environmental factors play an important role in disease prevention. In our study handwashing habits did not differ between the seronegative and seropositive groups. Although the literature identifies handwashing habits as a risk factor, our finding may not align with this expectation, likely because handwashing information was obtained through self-report. Moreover, given the high seroprevalence, widespread exposure to the virus may have overshadowed the measurable effect of personal hygiene in this particular cross-sectional snapshot. On the other hand, attending kindergarten was significantly higher in the seropositive group, highlighting the role of close contact in transmission. In logistic regression analysis, age, average monthly household income, and mother’s education level were found to be factors influencing seropositivity.

Within the scope of personal prevention, vaccine research is ongoing. Effective results have been reported with the EV-A71 vaccine, which was introduced in China in 2015 [33,34]. The overall EV-A71 vaccine effectiveness was reported to be 90.8% for all cases, and it reached 100% against severe cases [33]. Ongoing research efforts are also directed toward the development of multivalent formulations, such as the EV-A71/CV-A16 bivalent vaccine, the EV-A71/CV-A6/CV-A10 trivalent vaccine, and the EV-A71/CV-A16/CV-A6/CV-A10 tetravalent vaccine [34].

Our study has some limitations. Since it is based on admissions to outpatient clinics rather than a population-based screening, caution is warranted when generalizing the findings to the broader population. However, the fact that the hospitals where the study was conducted are located in five major cities and serve about a third of the country’s population (36.25%) is valuable in terms of representativeness. The exclusion of patients with chronic illnesses such as autoimmune diseases or immunodeficiency/immunosuppression may also have contributed to a bias. However, our study is valuable as it represents the first large-scale seroprevalence investigation conducted in Türkiye. We believe that our findings regarding circulating HFMD serotypes will serve as a guide in shaping prevention strategies.

## 5. Conclusions

The seroprevalence of HFMD-causing viruses in Türkiye is high from six months of age onward, underscoring the urgent need for nationwide preventive strategies. Nationwide strategies should be developed to address associated risk factors. To reduce transmission, policymakers should prioritize strengthening routine surveillance systems, integrating seroepidemiological data into public health planning, and promoting hygiene education programs in schools and childcare facilities. In addition to promoting personal protective measures, the implementation of a vaccination program should also be considered as part of a comprehensive national strategy. These measures would contribute to reducing the burden of HFMD in the population.

## Figures and Tables

**Table 1:** General characteristics of participants.

Study City	Ankara N = 194	Antalya N = 198	Adana N = 206	İzmir N = 181	İstanbul N = 219	Total N = 998
Age, year, mean ± SD (%95 CI)	8.08 ± 5.41 (7.31–8.84)	7.79 ± 5.12 (7.07–8.50)	8.95 ± 5.29 (8.22–9.68)	7.77 ± 5.27 (6.99–8.54)	10.10 ± 4.70 (9.48–10.73)	8.59 ± 5.22 (8.26–8.91)
Age Group, n (%)						
6 months–2 years	46 (23.7)	45 (22.7)	36 (17.5)	44 (24.3)	19 (8.7)	191 (19.0)
3–5 years	38 (19.6)	44 (22.2)	39 (18.9)	33 (18.2)	33 (15.1)	188 (18.7)
6–11 years	55 (28.4)	59 (29.8)	60 (29.1)	57 (31.5)	74 (33.8)	305 (30.6)
12–17 years	55 (28.4)	50 (25.3)	71 (34.5)	47 (26.0)	93 (42.5)	316 (31.7)
Sex, n (%)						
Male	111 (57.2)	104 (52.5)	111 (53.9)	97 (53.6)	89 (40.6)	512 (51.3)
Female	83 (42.8)	94 (47.5)	95 (46.1)	84 (46.4)	130 (59.4)	486 (48.7)

**Table 2:** Viral antibody test results by age group.

	Negative	Positive				Total	
		Single	Dual	Triple	Quadruple		
Age group	n (%)	n (%)	n (%)	n (%)	n (%)	n (%)	*p*
6–8 months	2 (9.5)	6 (28.6)	7 (33.3)	5 (23.8)	1 (4.8)	21 (100.0)	<0.001
9–11 months	2 (8.7)	4 (17.4)	7 (30.4)	8 (34.8)	2 (8.7)	23 (100.0)	
12–14 months	5 (20.8)	7 (29.2)	6 (25.0)	3 (12.5)	3 (12.5)	24 (100.0)	
15–17 months	0 (0.0)	11 (50.0)	9 (40.9)	2 (9.1)	0 (0.0)	22 (100.0)	
18–20 months	3 (11.1)	7 (25.9)	9 (33.3)	6 (22.2)	2 (7.4)	27 (100.0)	
21–24 months	2 (11.8)	3 (17.6)	7 (41.2)	4 (23.5)	1 (5.9)	17 (100.0)	
25–35 months	5 (8.9)	13 (23.2)	16 (28.6)	16 (28.6)	6 (10.7)	56 (100.0)	
3–5 years	16 (8.6)	46 (24.6)	46 (24.6)	52 (27.8)	27 (14.4)	187 (100.0)	
6–11 years	15 (4.9)	51 (16.7)	79 (25.9)	99 (32.5)	61 (20.0)	305 (100.0)	
12–17 years	3 (0.9)	32 (10.1)	66 (20.9)	135 (42.7)	80 (25.3)	316 (100.0)	

**Table 3:** Serotypes by age group and study sites.

		Serotypes			
		CV-A16	EV-A71	CV-A6	CV-A10
Age Group	N	n (%)	n (%)	n (%)	n (%)
6 months–2 years	190	84 (44.2)	121 (63.7)	82 (43.2)	78 (41.1)
3–5 years	187	92 (49.2)	109 (58.3)	110 (58.8)	91 (48.7)
6–11 years	305	140 (45.9)	202 (66.2)	223 (73.1)	187 (61.3)
12–17 years	316	143 (45.3)	232 (73.4)	269 (85.1)	245 (77.5)
*p*		0.777	0.004	<0.001	<0.001
Study Sites	N	n (%)	n (%)	n (%)	n (%)
Ankara	194	89 (45.9)	144 (74.2)	124 (63.9)	114 (58.8)
Antalya	198	80 (40.4)	145 (73.2)	142 (71.7)	126 (63.6)
Adana	206	137 (66.5)	127 (61.7)	152 (73.8)	122 (59.2)
İzmir	181	100 (55.2)	131 (72.4)	127 (70.2)	110 (60.8)
İstanbul	219	53 (24.2)	117 (53.4)	139 (63.5)	129 (58.9)
*p*		<0.001	<0.001	0.084	0.846

**Table 4:** Baseline characteristics and medical history stratified by test result.

	All-Negative (N = 53)	Any-Positive (N = 945)	OR (95% CI)	*p*
	n (%)	n (%)		
Age group				
6–8 months	2 (3.8)	19 (2.0)		<0.001 *
9–11 months	2 (3.8)	21 (2.2)	1.10 (0.14–8.63)	
12–14 months	5 (9.4)	19 (2.0)	0.39 (0.06–2.32)	
15–17 months	0 (0.0)	22 (2.3)	-	
18–20 months	3 (5.7)	24 (2.5)	0.84 (0.12–5.56)	
21–24 months	2 (3.8)	15 (1.6)	0.78 (0.09–6.27)	
25–35 months	5 (9.4)	51 (5.4)	1.07 (0.19–6.01)	
3–5 years	16 (30.2)	171 (18.1)	1.12 (0.24–5.27)	
6–11 years	15 (28.3)	290 (30.7)	2.03 (0.43–9.55)	
12–17 years	3 (5.7)	313 (33.1)	10.9 (1.73–69.7)	
Routine vaccination status by age				
Complete	44 (83.0)	892 (94.5)		0.003 *
Incomplete	9 (17.0)	41 (4.3)	4.45 (2.03–9.73)	
Unknown	0 (0.0)	11 (1.2)	-	
Daily handwashing				
Never	2 (4.1)	11 (1.2)		0.329 *
Very rarely	15 (30.6)	311 (35.2)	3.76 (0.76–18.5)	
Rarely	23 (46.9)	427 (48.4)	3.37 (0.70–16.1)	
Occasionally	9 (18.4)	134 (15.2)	2.70 (0.51–14.1)	
Handwashing after playtime				
Never	7 (13.2)	130 (13.8)		1.000 *
Sometimes	31 (58.5)	541 (57.2)	0.93 (0.40–2.18)	
Always	15 (28.3)	267 (28.3)	0.95 (0.38–2.40)	
Unknown	0 (0.0)	7 (0.7)	-	
Handwashing before meals				
Never	7 (13.2)	89 (9.4)		0.692 *
Sometimes	26 (49.1)	497 (52.6)	1.50 (0.63–3.56)	
Always	20 (37.7)	356 (37.7)	1.39 (0.57–3.41)	
Unknown	0 (0.0)	3 (0.3)	-	
Total number of household members				
1–3	17 (32.7)	211 (22.6)		0.221 *
4–6	34 (65.4)	690 (74.0)	1.63 (0.89–2.98)	
7 or more	1 (1.9)	32 (3.4)	2.57 (0.33–20.0)	
Household members aged 0–5 years	29 (55.8)	484 (51.9)	0.85 (0.48–1.49)	0.584 **
Household members aged 6–11 years	27 (51.9)	605 (64.8)	1.70 (0.97–2.98)	0.060 **
Household members aged 12–18 years	11 (21.2)	415 (44.5)	2.98 (1.51–5.88)	0.001 **
Mother’s education level				
Primary education	13 (24.5)	412 (43.6)		0.048 *
High school graduate	25 (47.2)	352 (37.2)	0.44 (0.22–0.88)	
Bachelor’s degree and above	14 (26.4)	174 (18.4)	0.39 (0.18–0.85)	
Illiterate	1 (1.9)	7 (0.7)	0.22 (0.02–1.92)	
Father’s education level				
Primary education	15 (28.3)	245 (25.9)		0.045 *
High school graduate	24 (45.3)	471 (49.8)	1.20 (0.61–2.33)	
Bachelor’s degree and above	13 (24.5)	229 (24.2)	1.07 (0.50–2.31)	
Illiterate	1 (1.9)	0 (0.0)	-	
Household’s average monthly income, TL				
<20,000	12 (22.6)	133 (14.1)		0.061 *
20,000–<50,000	21 (39.6)	279 (29.6)	1.19 (0.57–2.50)	
50,000–<75,000	13 (24.5)	341 (36.2)	2.36 (1.05–5.31)	
≥75,000	7 (13.2)	190 (20.1)	2.44 (0.93–6.38)	
Any history of HFMD	3 (5.7)	124 (13.1)	2.51 (0.77–8.19)	0.113 **
Any history of other rash illnesses	13 (24.5)	196 (20.7)	0.82 (0.43–1.57)	0.640 **
Nursery attendance	10 (18.9)	235 (24.9)	1.42 (0.70–2.87)	0.323 **
Kindergarten attendance	11 (20.8)	432 (45.7)	3.21 (1.63–6.32)	<0.001 **
Kindergarten attendance without prior nursery	4 (9.3)	266 (37.5)	5.84 (2.06–16.5)	<0.001 **

* Fisher Exact Test; ** Chi-Square Test. HFMD: Hand, foot and mouth disease.

**Table 5:** Factors influencing seropositivity-logistic regression analysis.

	OR (95% CI)	*p*
Age	1.177 (1.098–1.262)	<0.001
Household’s average monthly income (<20,000 TL)	-	
Household’s average monthly income (20,000–<50,000 TL)	1.336 (0.605–2.948)	0.474
Household’s average monthly income (50,000–<75,000 TL)	4.056 (1.667–9.872)	0.002
Household’s average monthly income (≥75,000–100,000)	5.640 (1.973–16.123)	0.001
Mother’s education level (Bachelor’s degree and above)	-	
Mother’s education level (High school graduate)	3.419 (1.479–7.907)	0.004
Mother’s education level (Primary education)	1.078 (0.521–2.230)	0.839

## Data Availability

Due to the provisions outlined in the informed consent form (ICF), the raw data are not publicly available. However, specific portions of the data may be shared upon reasonable request to the corresponding author, subject to appropriate justification and approval.

## Supplementary Materials

The following supporting information can be downloaded at: https://www.mdpi.com/article/10.3390/vaccines14060470/s1, Supplementary File S1: Case Report Form of the Study in Turkish.

## Abbreviations

The following abbreviations are used in this manuscript:

HFMD	Hand, foot and mouth disease
EV-A71	Enterovirus type 71
CV-A16	Coxsackievirus A16
CV-A10	Coxsackievirus A10
CV-A6	Coxsackievirus
EUROSTAT	The Statistical Office of the European Union
NUTS	The Nomenclature of Territorial Units for Statistics
HIV	Human Immunodeficiency Virus
CI	Confidence Interval
SD	Stanadard Deviation
IgG	Immunoglobulin G

## Appendix A

**Table A1:** Demographic, climatic, and socio-economic characteristics of study sites.

City	Ankara	Antalya	Adana	İzmir	İstanbul
Study site	Ankara Bilkent City Hospital	Akdeniz University Medical Faculty Hospital	Adana City Hospital	İzmir City Hospital	Prof. Dr. Cemil Taşcıoğlu City Hospital
EUROSTAT NUTS-1 Region	TR5-West Anatolia	TR6-Mediterranean	TR6-Mediterranean	TR3-Aegean	TR1-İstanbul
Temperature-Mean, January	0.3 °C	10.6 °C	10.0 °C	8.8 °C	5.5 °C
Temperature-Mean, July	23.4 °C	28.0 °C	28.1 °C	27.0 °C	23.6 °C
General population by city 2024	5,864,049	2,722,103	2,280,484	4,493,242	15,701,602
Population <18 y by city 2024	1,332,868	643,299	628,383	931,178	3,714,755
Population <12 y by city 2024	845,530	409,861	404,984	593,953	2,394,700
Percentage of population at risk for HFMD (<18 y)	22.7%	23.6%	27.6%	20.7%	23.7%
Socio-Economic status	Developed	Touristic	Agricultural	Developed	Dynamic

**Table A2:** Serotypes distribution by age groups and study sites.

	Ankara		Antalya		Adana		İzmir		İstanbul	
	N	n (%)	N	n (%)	N	n (%)	N	n (%)	N	n (%)
CV-A16										
6–8 months	8	3 (37.5)	3	2 (66.7)	3	2 (66.7)	4	2 (50.0)	3	0 (0.0)
9–11 months	8	3 (37.5)	6	2 (33.3)	1	0 (0.0)	8	5 (62.5)	0	0 (0.0)
12–14 months	1	0 (0.0)	8	5 (62.5)	1	0 (0.0)	11	5 (45.5)	3	0 (0.0)
15–17 months	4	2 (50.0)	5	3 (60.0)	6	3 (50.0)	5	0 (0.0)	2	0 (0.0)
18–20 months	7	5 (71.4)	5	1 (20.0)	7	5 (71.4)	5	2 (40.0)	3	0 (0.0)
21–24 months	4	1 (25.0)	4	2 (50.0)	5	4 (80.0)	0	0 (0.0)	4	0 (0.0)
25–35 months	14	8 (57.1)	14	7 (50.0)	13	7 (53.8)	11	5 (45.5)	4	0 (0.0)
3–5 years	38	21 (55.3)	44	12 (27.3)	39	26 (66.7)	33	22 (66.7)	33	11 (33.3)
6–11 years	55	25 (45.5)	59	23 (39.0)	60	42 (70.0)	57	31 (54.4)	74	19 (25.7)
12–17 years	55	21 (38.2)	50	23 (46.0)	71	48 (67.6)	47	28 (59.6)	93	23 (24.7)
EV-A71										
6–8 months	8	8 (100.0)	3	1 (33.3)	3	3 (100.0)	4	2 (50.0)	3	2 (66.7)
9–11 months	8	6 (75.0)	6	3 (50.0)	1	1 (100.0)	8	6 (75.0)	0	0 (0.0)
12–14 months	1	1 (100.0)	8	5 (62.5)	1	1 (100.0)	11	4 (36.4)	3	0 (0.0)
15–17 months	4	3 (75.0)	5	1 (20.0)	6	3 (50.0)	5	3 (60.0)	2	2 (100.0)
18–20 months	7	7 (100.0)	5	4 (80.0)	7	5 (71.4)	5	4 (80.0)	3	2 (66.7)
21–24 months	4	4 (100.0)	4	2 (50.0)	5	1 (20.0)	0	0 (0.0)	4	2 (50.0)
25–35 months	14	8 (57.1)	14	9 (64.3)	13	5 (38.5)	11	10 (90.9)	4	3 (75.0)
3–5 years	38	17 (44.7)	44	35 (79.5)	39	19 (48.7)	33	27 (81.8)	33	11 (33.3)
6–11 years	55	43 (78.2)	59	43 (72.9)	60	44 (73.3)	57	41 (71.9)	74	31 (41.9)
12–17 years	55	47 (85.5)	50	42 (84.0)	71	45 (63.4)	47	34 (72.3)	93	64 (68.8)
CV-A6										
6–8 months	8	4 (50.0)	3	2 (66.7)	3	0 (0.0)	4	1 (25.0)	3	1 (33.3)
9–11 months	8	5 (62.5)	6	2 (33.3)	1	1 (100.0)	8	4 (50.0)	0	0 (0.0)
12–14 months	1	0 (0.0)	8	6 (75.0)	1	0 (0.0)	11	5 (45.5)	3	1 (33.3)
15–17 months	4	0 (0.0)	5	3 (60.0)	6	2 (33.3)	5	3 (60.0)	2	0 (0.0)
18–20 months	7	4 (57.1)	5	0 (0.0)	7	2 (28.6)	5	1 (20.0)	3	1 (33.3)
21–24 months	4	4 (100.0)	4	2 (50.0)	5	4 (80.0)	0	0 (0.0)	4	0 (0.0)
25–35 months	14	5 (35.7)	14	6 (42.9)	13	7 (53.8)	11	5 (45.5)	4	1 (25.0)
3–5 years	38	20 (52.6)	44	31 (70.5)	39	25 (64.1)	33	21 (63.6)	33	13 (39.4)
6–11 years	55	40 (72.7)	59	44 (74.6)	60	47 (78.3)	57	44 (77.2)	74	48 (64.9)
12–17 years	55	42 (76.4)	50	46 (92.0)	71	64 (90.1)	47	43 (91.5)	93	74 (79.6)
CV-A10										
6–8 months	8	4 (50.0)	3	1 (33.3)	3	0 (0.0)	4	0 (0.0)	3	1 (33.3)
9–11 months	8	5 (62.5)	6	3 (50.0)	1	1 (100.0)	8	3 (37.5)	0	0 (0.0)
12–14 months	1	0 (0.0)	8	6 (75.0)	1	0 (0.0)	11	1 (9.1)	3	0 (0.0)
15–17 months	4	1 (25.0)	5	2 (40.0)	6	1 (16.7)	5	3 (60.0)	2	0 (0.0)
18–20 months	7	4 (57.1)	5	1 (20.0)	7	0 (0.0)	5	2 (40.0)	3	1 (33.3)
21–24 months	4	4 (100.0)	4	1 (25.0)	5	1 (20.0)	0	0 (0.0)	4	1 (25.0)
25–35 months	14	8 (57.1)	14	11 (78.6)	13	3 (23.1)	11	7 (63.6)	4	2 (50.0)
3–5 years	38	14 (36.8)	44	27 (61.4)	39	20 (51.3)	33	20 (60.6)	33	10 (30.3)
6–11 years	55	33 (60.0)	59	35 (59.3)	60	39 (65.0)	57	41 (71.9)	74	39 (52.7)
12–17 years	55	41 (74.5)	50	39 (78.0)	71	57 (80.3)	47	33 (70.2)	93	75 (80.6)
