# Supplementary material for: Seroprevalence of Hand, Foot and Mouth Disease Among Children and Adolescents in Türkiye
Source: Vaccines (Basel). 2026 May 25;14(6):470. doi: 10.3390/vaccines14060470 (PMC13307745; doi:10.3390/vaccines14060470)
Supplement: Supplementary file 1 [file vaccines-14-00470-s001.zip › vaccines-4268374-supplementary.pdf]

Veri Toplama Formu-Bölüm A

Merkez no: __________

Vizit Tarihi: __________ / __________ / __________ (gg/aa/yyyy)

Gönüllü No: A-☐☐-☐☐☐

ÇALIŞMA BARKODU

Gönüllü çalışmaya alınmaya uygun mu? ☐ Evet ☐ Hayır

I. Temel Bilgiler

1. İsmin baş harfleri: __________ / __________ / __________

2. Cinsiyet: ☐ Erkek ☐ Kadın

3. Doğum tarihi: __________ / __________ / __________ (gg/aa/yyyy)

4. Yaşanılan yer: ☐ Kırsal ☐ Şehir

5. Kreşe gitme durumu: ☐ Gitmedi ☐ Gitti ➔Başlama Yaşı: ...... ve Gittiği Süre: .... yıl

6. Anaokuluna gitme durumu: ☐ Gitmedi ☐ Gitti ➔Başlama Yaşı: ...... ve Gittiği Süre: .... yıl

7. Okul durumu: ☐ Hayır ☐ İlkokul ☐ Lise ☐ Bilinmiyor

8. Yaşa göre rutin aşılama: ☐ Tam ☐ Eksik ☐ Bilinmiyor

9. Günlük el yıkama sayısı: ....

10. Oyun sonrası el yıkama: ☐ Hiç ☐ Bazen ☐ Her zaman ☐ Bilinmiyor

Hastane/Merkez: .... Doktor/Araştırmacı: .... Rapor tarihi: ....

Veri Toplama Formu-Bölüm A

11. Yemekten önce el yıkama: ☐ Hiç ☐ Bazen ☐ Her zaman ☐ Bilinmiyor

12. Aynı hanede yaşayan toplam aile üyesi sayısı: ....

0-5 yaş aile üyesi sayısı: __________
6-11 yaş aile üyesi sayısı: __________
12-18 yaş aile üyesi sayısı: __________

13. Annenin eğitimi: ☐ İlköğretim ☐ Lise mezunu ☐ ≥ Lisans düzeyi

14. Babanın eğitimi: ☐ İlköğretim ☐ Lise mezunu ☐ ≥ Lisans düzeyi

15. Ailenin ortalama aylık geliri: ☐ <20.000 TL ☐ <50.000 TL

☐ <75.000 TL ☐ 75.000 TL - 100.000 TL

II. Tıbbi Öykü

16. Herhangi bir EAAH öyküsü? ☐ Hayır ☐ Evet

Evet ise, başlangıç tarihi: __________ / ___________ (aa/yyyy) ☐ Tarih bilinmiyor

Evet ise, hastaneye yatırılma ☐ Hayır ☐ Evet ☐ Bilinmiyor

17.Başka bir döküntü öyküsü? ☐ Hayır ☐ Evet ☐ Bilinmiyor

18. Hastaneye şimdiki başvuru nedeniniz? Lütfen nedenini aşağıda belirtin:

____

____

19. Fiziksel muayene (İsteğe bağlı, vizitte yapılmışsa):

Nabız: .... Vücut ısısı (koltuk altı) (℃): ....

III. Numune Alımı

20. Kan örneği: ☐ Hayır ☐ Evet

Hastane/Merkez: .... Doktor/Araştırmacı: .... Rapor tarihi: ....
